# Supplying the sleeping brain

**DOI:** 10.7554/eLife.64597

**Published:** 2020-12-23

**Authors:** Stephanie D Williams, Laura D Lewis

**Affiliations:** 1 Department of Psychological and Brain Sciences, Boston UniversityBostonUnited States; 2 Department of Biomedical Engineering, Boston UniversityBostonUnited States

**Keywords:** neurovascular coupling, sleep, optical imaging, electrophysiology, blood flow, arousal state, Mouse

## Abstract

During sleep, the brain experiences large fluctuations in blood volume and altered coupling between neural and vascular signals.

**Related research article** Turner KL, Gheres KW, Proctor EA, Drew PJ. 2020. Neurovascular coupling and bilateral connectivity during NREM and REM sleep. *eLife* **9**:e62071. doi: 10.7554/eLife.62071

The amount of blood the brain receives is tightly regulated to respond to the metabolic needs of neurons. This ‘neurovascular coupling’ allows flexible delivery of energy, with blood rushing in when neurons are active (Iadecola, 2017). Many brain imaging techniques rely on this process. Functional magnetic resonance imaging (or fMRI for short), in particular, uses blood oxygenation signals to pinpoint changes in neural activity. Understanding neurovascular dynamics is therefore essential for neuroscience research and the correct interpretation of fMRI measurements.

Sleep profoundly transforms most aspects of brain physiology, including blood oxygenation signals (Horovitz et al., 2008), but its effects on neurovascular dynamics are not well understood. Now, in eLife, Patrick J. Drew and colleagues from Pennsylvania State University – with Kevin Turner as first author – report an in-depth characterization of how blood volume and neurovascular coupling change across arousal states in mice (Turner et al., 2020; Figure 1).

**Figure 1.**
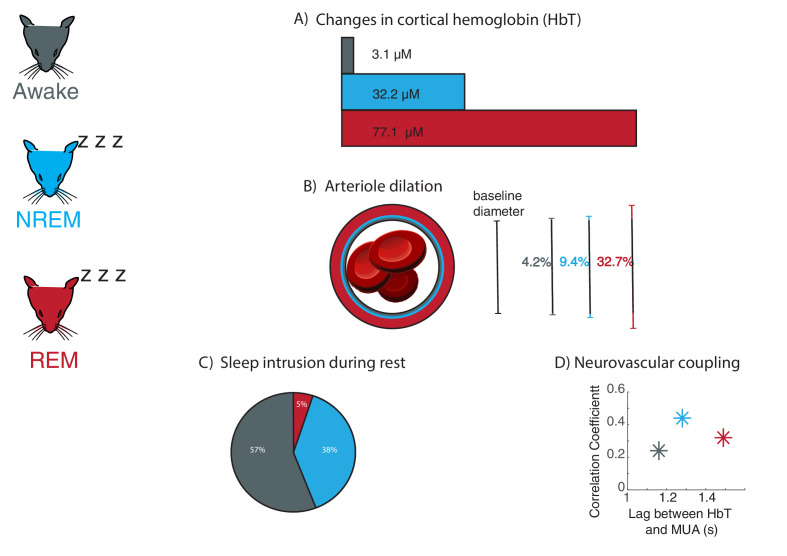
Large fluctuations in blood volume and altered neurovascular coupling occur during sleep in mice. Changes in neurovascular dynamics were examined in head-fixed mice (left) which were awake (grey), in non-rapid eye movement sleep (NREM; blue), and rapid eye movement sleep (REM; red). (**A**) As inferred from changes in cortical hemoglobin levels, total blood volume increases drastically during sleep, especially during REM sleep. (**B**) Average arteriole dilation during sleep increases above the level found during the awake state. (**C**) Sleep occurs during eyes-open behavioral rest in head-fixed mice. NREM sleep (38% of rest periods) commonly occurred during behavioral rest. REM sleep was less common but still occurred (5% of rest periods). (**D**) The strength of coupling between neural signals (multi-unit activity, or MUA) and levels of cortical hemoglobin (HbT) increased during NREM sleep, and the timing of coupling shifted across sleep stages.

Image credit: Mouse illustrations adapted from Luigi Petrucco via SciDraw (CCBY 4.0); Blood cells illustrations adapted from John Chilton via SciDraw (CCBY 4.0); Panel C adapted from Turner et al., 2020 (CCBY 4.0).

To investigate this question, Turner et al. monitored behavioral, neural, and vascular signals while mice were awake or in non-rapid and rapid eye movement sleep (respectively NREM and REM). The heads of the animals were immobilized to allow imaging. However, mice can still sleep with their eyes open under these conditions, prompting Turner et al. to collect detailed behavioral and physiological measurements to determine sleep stages.

First, intrinsic optical signal imaging was used to infer the amount of hemoglobin, which in turn is correlated with the volume of blood in the brain. This method relies on the light reflection changing with the hemoglobin content, and it revealed large variations in the volume of blood in the brain during sleep. When mice entered NREM sleep, the amplitude of oscillations in hemoglobin levels more than doubled compared to the awake state; during REM sleep, they showed a prolonged increase in total hemoglobin that lasted more than 30 seconds. The amount of total hemoglobin changed drastically as mice woke up or fell asleep, highlighting that blood volume dynamics are coupled with arousal state. Together, these results show that blood volume in the brain fluctuates greatly during sleep.

In addition to these large-scale measurements of hemoglobin, Turner et al. tracked the diameter of tiny vessels known as arterioles. During NREM sleep, arteriole diameters fluctuated greatly, and they increased even further (over tens of seconds) when mice entered REM sleep. These measurements helped to unravel how arterioles, capillaries and veins each contribute to the changes in global blood volume measured by intrinsic optical signal imaging.

Next, Turner et al. examined whether the changes in blood volume during sleep were linked to variations in neural activity, which normally drive fluctuations in the diameter of arterioles (Mateo et al., 2017). This revealed that the strength of the neurovascular coupling changed depending on arousal states, increasing substantially during NREM sleep compared to wakefulness. The timing of the coupling was also altered, with the blood volume response being slower during REM sleep than in the awake state.

Many studies that use head-fixed mice do not systemically assess whether the animals are awake, raising the question of how much time they actually spend asleep. Analyzing all 15 seconds rest periods in which mice had their eyes open showed that they were actually sleeping in nearly half of these intervals. In addition, only measuring heart rate or whisker movements could not reliably identify sleeping periods. These findings suggest that head-fixed imaging studies should include measurements of arousal state to detect if the mice are asleep.

Overall, Turner et al. identify a striking transformation of neurovascular dynamics during sleep: blood volume increases and exhibits large oscillations, and coupling to neural activity is altered. Intriguingly, vascular dynamics during sleep, including variations in blood volume, are much larger than those driven by the well-studied stimuli and behaviors found in the awake state. Why is this the case? In addition to changes in neural activity and cognition, sleep also involves increased waste clearance from the brain, which has been linked to vascular oscillations (Xie et al., 2013; Iliff et al., 2013; Mestre et al., 2018; van Veluw et al., 2020). Thus, these results also help identify the consequences of sleep for the brain, and how blood dynamics may contribute to brain health.
